# Up‐regulated TLR4 in cardiomyocytes exacerbates heart failure after long‐term myocardial infarction

**DOI:** 10.1111/jcmm.12659

**Published:** 2015-08-20

**Authors:** Li Liu, Yin Wang, Zhi‐Yong Cao, Meng‐Meng Wang, Xue‐Mei Liu, Ting Gao, Qi‐Kuan Hu, Wen‐Jun Yuan, Li Lin

**Affiliations:** ^1^ Department of Physiology and Key Lab of Ministry of Education in Fertility Preservation and MaintenanceNingxia Medical UniversityYinchuanChina; ^2^ Department of UltrasonographyChanghai HospitalShanghaiChina; ^3^ Department of General Internal MedicineBranch of 411 Hospital of People's Liberation ArmyShanghaiChina; ^4^ Department of PhysiologySecond Military Medical UniversityShanghaiChina; ^5^ Key Laboratory of Arrhythmias of the Ministry of Education of ChinaShanghai East HospitalTongji University School of MedicineShanghaiChina

**Keywords:** heart failure, inflammation, toll‐like receptor, cardiomyocyte

## Abstract

It remains unclear whether and how cardiomyocytes contribute to the inflammation in chronic heart failure (CHF). We recently reviewed the capacity of cardiomyocytes to initiate inflammation, by means of expressing certain immune receptors such as toll‐like receptors (TLRs) that respond to pathogen‐ and damage‐associated molecular patterns (PAMP and DAMP). Previous studies observed TLR4‐mediated inflammation within days of myocardial infarction (MI). This study examined TLR4 expression and function in cardiomyocytes of failing hearts after 4 weeks of MI in rats. The increases of TLR4 mRNA and proteins, as well as inflammatory cytokine production, were observed in both the infarct and remote myocardium. Enhanced immunostaining for TLR4 was observed in cardiomyocytes but not infiltrating leucocytes. The injection of lentivirus shRNA against TLR4 into the infarcted heart decreased inflammatory cytokine production and improved heart function *in vivo*. Accordingly, in cardiomyocytes isolated from CHF hearts, increases of TLR4 mRNA and proteins were detected. More robust binding of TLR4 with lipopolysaccharide (LPS), a PAMP ligand for TLR4, and heat shock protein 60 (HSP60), a DAMP ligand for TLR4, was observed in CHF cardiomyocytes under a confocal microscope. The maximum binding capacity (B_max_) of TLR4 was increased for LPS and HSP60, whereas the binding affinity (Kd) was not significantly changed. Furthermore, both LPS and HSP60 induced more robust production of inflammatory cytokines in CHF cardiomyocytes, which was reduced by TLR4‐blocking antibodies. We conclude that the expression, ligand‐binding capacity and pro‐inflammatory function of cardiomyocyte TLR4 are up‐regulated after long‐term MI, which promote inflammation and exacerbate heart failure.

## Introduction

Chronic heart failure (CHF) is characterized by ongoing systemic inflammation that correlates with disease severity. Raised circulating levels of a portfolio of inflammatory cytokines such as tumour necrosis factor α (TNF‐α) and interleukin (IL)‐6 have been reported in patients with CHF 1, 2. Transcardiac increases in inflammatory cytokines and enhanced expression of inflammatory cytokines in the failing myocardium both demonstrate inflammation in the failing heart 3, 4, 5. Virtually all the cell types within the failing heart, including cardiomyocytes, fibroblasts, endothelial cells and infiltrating leucocytes, may contribute to the myocardial inflammation 1, 6. As the key cell type in the heart, cardiomyocytes are a potential source, as well as a target, of inflammation 7. However, the mechanisms that trigger and/or exacerbate inflammation in cardiomyocytes during the progression of CHF remain unclear.

Cardiac inflammation can be roughly distinguished as manifestations of either innate immune responses alone or a combination of innate and adaptive immune responses 8. Innate immune responses are characterized by the induction of inflammatory cytokines. Ischaemic heart disease, the leading cause of heart failure, is commonly involved by innate immune responses and inflammation. Toll‐like receptors (TLRs) are an important family of pattern recognition receptors (PRRs) that provoke innate immune responses. They are typically activated by pathogen‐associated molecular patterns (PAMPs) derived from microbial pathogens and damage‐associated molecular patterns (DAMPs) derived from damaged host cells 6, 9. So far ten functional TLRs have been identified in humans. Among them, TLR 1/2/4/5/6 are expressed on the cell surface, whereas TLR3/7/8/9 are localized in intracellular vesicles such as the endoplasmic reticulum, endosomes, lysosomes and endolysosomes 10. The TLRs predominantly expressed in cardiomyocytes are TLR2, TLR3, and TLR4 6. Based on the recruited adaptor proteins, TLR signalling can be largely divided to two pathways, respectively dependent on myeloid differentiation factor 88 (MyD88) and Toll/IL‐1 receptor (TIR) domain‐containing adaptor protein inducing interferon‐β (Trif). The MyD88‐dependent pathway can be activated by all TLRs except for TLR3. The Trif‐dependent pathway can be activated by TLR3 and TLR4. These two pathways converge on the activation of nuclear factor‐κB (NF‐κB), a key transcription factor for inflammatory activation 10.

Cumulative data demonstrate that TLRs play critical roles in mediating inflammatory responses associated with heart diseases including myocardial infarction (MI) 9, 11. However, little is known with respect to the functional status of TLRs in the failing heart, although TLR4 expression appears to be up‐regulated in the failing human heart 12, 13. The most common cause of CHF is ischaemic heart disease. The literature reports that TLR4 expression increases within days of MI 12, 14. We previously observed enhanced TLR4 expression after short‐term ischaemia in cultured cardiomyocytes, as well as intact heart 15. The chronic effects of ischaemia on the expression and function of TLR4 in cardiomyocytes remain unclear. There is a possibility that TLR4 changes may render cardiomyocytes to act as innate immune cells and initiate inflammation, as we discussed in a recent review 7. The present study examined this possibility in a rat model of CHF after 4 weeks of MI. By isolating cardiomyocytes from the failing heart, we examined the mRNA and protein expression of TLR4 in cardiomyocytes, and its binding activity and inflammatory responses to PAMP and DAMP ligands.

## Materials and methods

### Rat model of CHF induced by myocardial infarction

A rat coronary ligation model of heart failure was prepared, as we described previously 16. Adult male Sprague–Dawley (SD) rats (3–4 months old, 260–330 g; SIPPR‐BK Laboratory Animal Co. Ltd, Shanghai, China) were intra‐peritoneally anaesthetized with ketamine (100 mg/kg) and xylazine (10 mg/kg), and maintained under anaesthesia with bolus injections of ketamine and xylazine as required. The adequacy of anaesthesia was checked by the lack of corneal reflex and withdrawal reflex to toe pinch. The rats were tracheotomized and artificially ventilated at a stroke volume of 1.5–2 ml/100 g and a rate of 60 strokes/min. A left thoracotomy was performed and MI was induced by ligating the left anterior descending coronary artery (LAD) with a 6/0 braided silk suture. MI was then confirmed by electrocardiography and visual cyanosis of the heart. Sham rats received the same procedure except that LAD was not ligated.

Four weeks after the surgery, the rats were killed with an overdose intra‐peritoneal injection of ketamine (300 mg/kg) and xylazine (30 mg/kg), the heart and blood samples were then collected for downstream experiments. Both the infracted and remote tissues were sampled from the left ventricle of LAD‐ligated rats for quantitative assays. Accordingly, anterior and posterior ventricular tissues were sampled from the sham rats to serve as controls, which were referred to as ‘sham‐infarct’ and ‘sham‐remote’ in Figures 2 and 3.

All animal procedures were approved by the Animal Experiment Committee of Ningxia Medical University, in accordance with the Guide for the Care and Use of Laboratory Animals published by the US National Institutes of Health (NIH Publication, 8th Edition, 2011).

### Preparation and application of lentivirus shRNA against TLR4

On the basis of an effective siRNA targeting TLR4 that we identified previously 15, we synthesized the following shRNA against TLR4: 5′‐TGCGAGCTGGTAAAGAATTTATTCAAGAGATAAATTCTTTACCAGCTCGCTTTTTTC‐3′ (sense) and 5′‐TCGAGAAAAAAGCGAGCTGGTAAAGAATTTATCTCTTGAATAAATTCTTTACCAGCTCGCA‐3′ (anti‐sense). A scrambled sequence of the same length was used as control: 5′‐TGTTCTCCGAACGTGTCACGTTTCAAGAGATAAATTCTTTACCAGCTCGCTTTTTTC‐3′ (sense) and 5′‐TCGAGAAAAAAGTTCTCCGAACGTGTCACGTTCTCTTGAATAAATTCTTTACCAGCTCGCA‐3′ (anti‐sense). The lentiviruses expressing either TLR4 shRNA or control shRNA were constructed, and confirmed by DNA sequencing. All lentiviruses were custom‐made by Shanghai GenePharma Co., Ltd, Shanghai, China.

For delivery of lentiviruses to the myocardium, approximately 100 μl/heart (1 × 10^9^ TU/ml) of TLR4‐shRNA lentivirus or control shRNA lentivirus was injected into the left ventricle at five sites around the infarct border, just after LAD ligation or sham operation. An equivalent volume of normal saline was injected as a control.

### Haematoxylin and eosin staining and Masson's trichrome staining

Haematoxylin and eosin staining and Masson's trichrome staining were performed to observe histopathological changes in the myocardium after infarction. Briefly, the heart was perfused with 4% paraformaldehyde, dehydrated with ethanol, embedded in paraffin blocks, sectioned into 5‐μm‐thick slices and stained with commercial reagents for haematoxylin and eosin and Masson's staining respectively (Guge Biotechnology Co., Ltd, Wuhan, China). In haematoxylin and eosin staining, nuclei were stained blue‐purple, and cytoplasm and extracellular matrix were stained varying shades of pink. In Masson's trichrome staining, collagen fibres were stained green‐blue, nuclei were stained dark and cardiac muscles were stained purple‐red.

### Infarct size measurement

Using Masson's trichrome staining photos, the infarct size was determined with a length‐based approach described by Takagawa *et al*. 17. In this approach, the midline infarct length was taken as the midline of the length of infarct that included greater than 50% of the whole thickness of the myocardial wall, and the infarct size (percentage of left ventricle) was calculated by dividing the midline infarct length by the LV midline circumference and multiplying by 100.

### Cardiac echo

The heart function was determined by transthoracic echocardiography 4 weeks after LAD ligation, using an ultrasonic apparatus (Voluson E8; GE Healthcare, Little Chalfont, Buckinghamshire, UK, 15‐MHz probe) 16. The rats were anaesthetized with ketamine (50 mg/kg) and xylazine (5 mg/kg), their chests were depilated, and the echocardiography was performed. The short‐axis view of the heart at the papillary muscle level was acquired by two‐dimensional imaging. Consecutive M‐mode images in the short‐axis view were recorded for analysis of chamber size and fractional shortening of the left ventricle.

### Isolation and culture of cardiomyocytes

Cardiomyocytes were isolated from sham and CHF rats and cultured, following a previous procedure 15. Hearts were removed from anaesthetized rats, mounted on a Langendorff system, and retrogradely digested with calcium‐free Krebs–Henseleit buffer containing 1 mg/ml collagenase type 2 (Worthington Biochemical, Lakewood, NJ, USA), saturated with 95% O_2_‐5% CO_2_ at 37°C. When the heart became flaccid, the ventricles were minced, and digested further in a shaking water bath. Dissociated cells were then collected, brought back to calcium‐containing buffer, pre‐plated to remove fibroblasts, and cultured with DMEM (Sigma‐Aldrich Corp., St. Louis, MO, USA) supplemented with 10% foetal bovine serum (HyClone Laboratories, Logan, UT, USA) in a CO_2_ incubator at 37°C. The cardiomyocytes were cultured for 24 hrs before downstream experiments, including TLR4 binding and function assay.

To determine the pro‐inflammatory function of TLR4, cultured cardiomyocytes were treated with the PAMP ligand lipopolysaccharide (LPS, Cat. L4524; Sigma‐Aldrich Corp.), or the DAMP ligand heat shock protein 60 (HSP60, low endotoxin, Cat. ADI‐ESP‐741; Enzo Life Sciences, Inc., Farmingdale, NY, USA). Toll‐like receptor 4 neutralizing antibody (anti‐TLR4) was added 15 min before the treatment of LPS or HSP60.

### TLR4 binding assay

The binding activity of TLR4 to LPS and HSP60 were determined as described previously 18. Cultured cardiomyocytes were incubated with FITC‐conjugated LPS (Cat. F3665, Sigma‐Aldrich Corp.) or Oregon Green 488‐labelled HSP60 (OG‐HSP60, prepared as below) at indicated concentrations for 30 min at 4°C, then the unbound ligand was washed away, and the cells were fixed and examined. The fluorescence image was observed under a confocal microscope (Leica, Heidelberg, Germany), and the fluorescence intensity was measured with a fluorescence microplate reader (BioTek, Winooski, VT, USA). To block TLR4, cultured cardiomyocytes were incubated with TLR4 neutralizing antibody (anti‐TLR4) at 37°C for 15 min., and subsequently incubated with FITC‐LPS or OG‐HSP60 at 4°C for 30 min.

OG‐HSP60 was prepared from commercial low‐endotoxin HSP60 (Cat. ADI‐ESP‐741; Enzo Life Sciences, Inc.) 18. The Oregon Green^®^ 488 isothiocyanate (F2FITC) mixed isomers (Molecular Probes, Life Technologies, Shanghai, China) were used to label HSP60, following the manufacturer's protocol. The concentration of prepared OG‐HSP60 was calculated by measuring the optical absorbance at 280 nm corrected by the absorbance for Oregon green and the extinction coefficient for HSP60 19.

### Real‐time RT‐PCR

Real‐time RT‐PCR was performed to determine the mRNA levels of TLR4, TNF‐α and IL‐6 in heart tissue and isolated cardiomyocytes. Total RNA was extracted with Trizol (Invitrogen, Shanghai, China), and reverse‐transcribed using M‐MLV reverse transcriptase with oligo‐dT. Real‐time quantitative PCR was performed on a Bio‐Rad MiniOpticon real‐time system (Bio‐Rad Laboratories, Inc., Hercules, CA, USA) using SYBR Green (Qiagen, Shanghai, China). All samples were analysed in duplicate. The 2^−∆Ct^ method was used to calculate the relative levels of target mRNA, and glyceraldehyde‐3‐phosphate dehydrogenase (GAPDH) was employed as an internal control. The PCR primers are listed in Table 1.

**Table 1:** Primers for real‐time PCR

Gene name	Accession no.	Forward primer (5′–3′)	Reverse primer (5′–3′)
TLR4	NM_019178.1	GCCGTCTTCAATCTGACTAAT	ACACTGACCACCGATACACT
TNF‐α	NM_012675.3	CCCAATCTGTGTCCTTCTAACT	CACTACTTCAGCGTCTCGTGT
IL‐6	NM_012589.1	GATTGTATGAACAGCGATGATG	CTCCAGGTAGAAACGGAACTC
GAPDH	NM_017008.3	AACGACCCCTTCATTGACCTC	CCTTGACTGTGCCGTTGAACT

TLR4, toll‐like receptor 4; TNF, tumour necrosis factor; IL, interleukin.

### Western blot analysis

Western blot was used to determine the protein levels of TLR4, as described previously 15. Heart tissue was homogenized and isolated cardiomyocytes were lysed in RIPA buffer supplemented with protease inhibitors (Beyotime Institute of Biotechnology, Jiangsu, China), sonicated on ice and protein concentration was determined using a bicinchoninic acid kit (Beyotime Institute of Biotechnology). The lysates (20 μg of total proteins) were electrophoresed on a 10% SDS‐PAGE gel and transferred onto a nitrocellulose membrane. The membrane was then blocked with 5% non‐fat dried milk, and probed with the primary antibody against TLR4 (Cat. NB100‐56566; Novus Biologicals, Littleton, CO, USA) followed by the peroxidase‐conjugated secondary antibody, at the concentration of 1:500 and 1:1000 respectively. The signal was visualized using chemiluminescence reagents, scanned with a GeneGnome Syngene Bio Imaging system and quantified by densitometry.

### Immunohisto‐ and immunocyto‐fluorescence staining

Heart samples embedded in paraffin were sectioned transversely at a thickness of 5 μm, mounted on gelatin‐coated glass slides, dried in an oven and stored at room temperature. Before staining, slides were deparaffinized/rehydrated, antigen retrieved by microwaving and blocked with 5% bovine serum albumin. Slides were then incubated overnight with primary antibodies against TLR4 (diluted 1:100, Cat. NB100‐56566; Novus Biologicals) and CD45 (diluted 1:50, Cat. ab10558; Abcam, Shanghai, China), a pan‐leucocyte marker and visualized with fluorescence‐labelled second antibodies. Similarly, isolated cardiomyocytes from sham and CHF rats were seeded on gelatine‐coated coverslips, and stained with anti‐TLR4 antibodies. Confocal microscopy was carried out using a Leica TCS SP5 microscope.

### ELISA analysis

The protein levels of TNF‐α and IL‐6 in heart tissue and isolated cardiomyocytes, as well as the serum levels of brain natriuretic peptide (BNP), TNF‐α and IL‐6, were measured using commercial ELISA kits (Xitang Biotech Co. Ltd, Shanghai, China), following the manufacturer's instructions.

### Determination of NF‐κB activation

The nuclear translocation of p65, a subunit of the NF‐κB heterodimer, was used as readout for NF‐κB activation. We examined p65 translocation by probing Western blots of nuclear extracts for p65 15.

### Statistics and data analysis

All the data are expressed as means ± SD, except that the maximum binding capacity (B_max_) and dissociation constant (Kd) are expressed as means ± SE. Differences between groups were determined by the one‐way anova followed by the Fisher's least significant difference test using SAS 9.0 statistical software (SAS Institute Inc., Cary, NC, USA). Ligand‐binding data were analysed by non‐linear regression with the one site‐specific binding option using Prism 5 software (GraphPad Software, Inc., San Diego, CA, USA). A *P *<* *0.05 was considered statistically significant.

## Results

### Inflammation in infarct and remote areas of the failing heart

**Figure 1:**
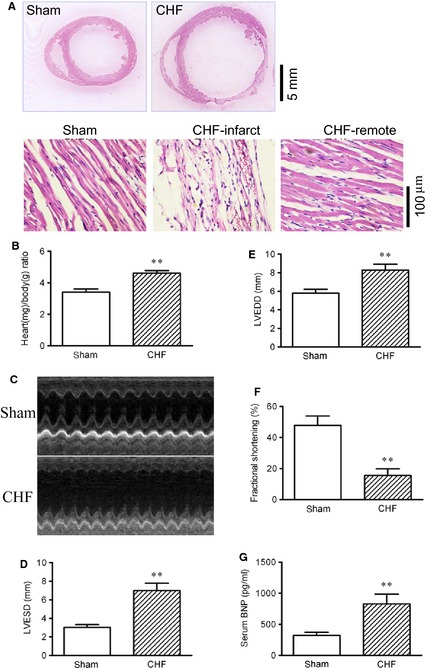
Myocardial infarction resulted in chronic heart failure (CHF) in rats after 4 weeks of coronary ligation. (**A**) Gross view and microscopic photos of heart sections stained with haematoxylin and eosin. Cross‐sections were cut at the midhorizontal plane of the fixed paraffin‐embedded heart, and stained with haematoxylin and eosin reagents. (**B**) Heart‐to‐body weight ratio. (**C**) Representative M‐mode ultrasound images of sham and CHF rats taken at the midpapillary level. (**D**) LV end‐systolic diameter (LVESD). (**E**) LV end‐diastolic diameter (LVEDD). (**F**) Fractional shortening (%) of the left ventricle. (**G**) Serum brain natriuretic peptide (BNP) level (data are means ± SD, n = 6–9/group, **P < 0.01 *versus* sham).

As expected, CHF occurred in the rats 4 weeks after coronary ligation. The haematoxylin and eosin staining showed marked wall thinning and fibrosis in the infarct area, and myocyte hypertrophy in the remote area (Fig. 1A). The infarct size in the present model was approximately 45%. Compared to sham‐operated rats, MI caused a significant increase in the heart‐to‐body weight ratio (Fig. 1B). In the echocardiograms, anterior wall motion abnormality was evident in all surviving rats subjected to MI (Fig. 1C). The two‐dimensional guided M‐mode ultrasound images taken at the midpapillary level showed significant increases in LV end‐systolic and end‐diastolic diameters, but a decrease in fractional shortening, indicating the dilatation of the left ventricle and decrease in LV contractility (Fig. 1D–F). Consistent with the decline in heart function, serum BNP levels increased after MI, also indicating the failure of the heart (Fig. 1G).

**Figure 2:**
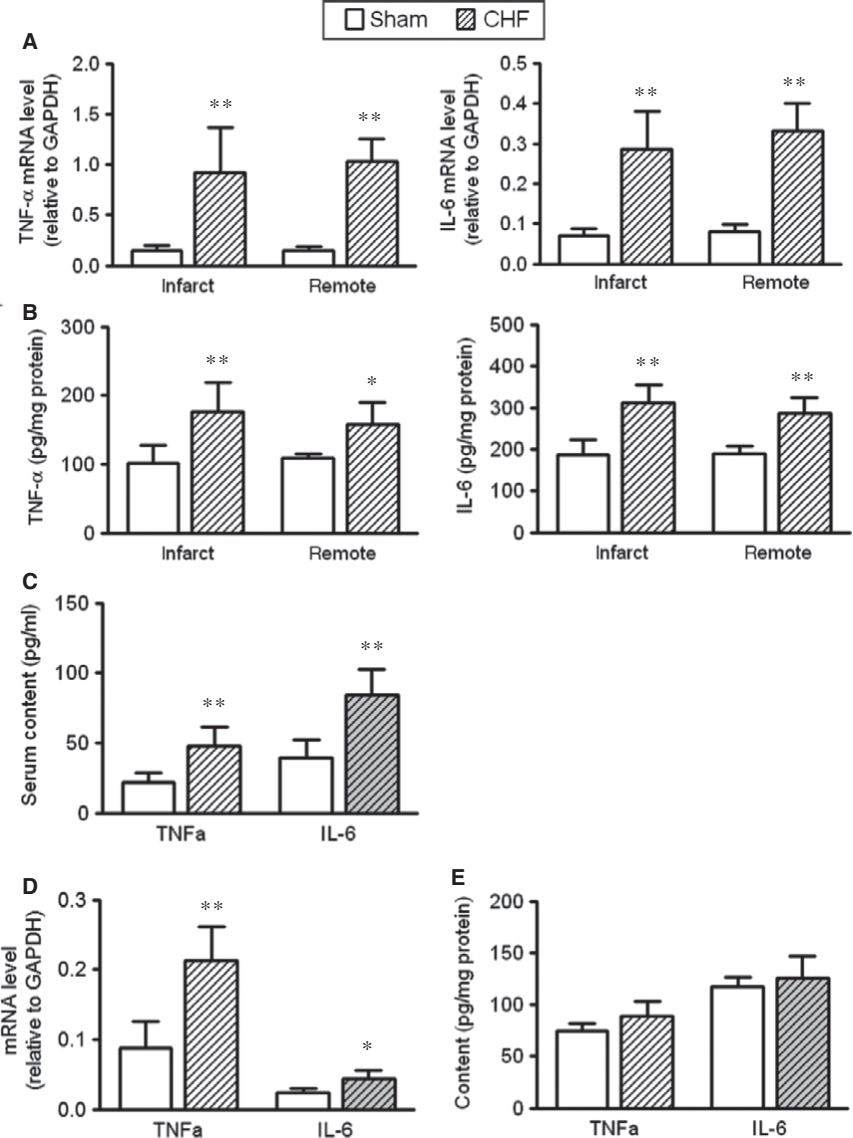
Inflammation in chronic heart failure (CHF) rats. (**A**) Tumour necrosis factor (TNF)‐α and interleukin (IL)‐6 mRNA levels in infarct and remote myocardium. (**B**) TNF‐α and IL‐6 protein levels in infarct and remote myocardium. (**C**) Serum content of TNF‐α and IL‐6 in sham and CHF rats. (**D**) TNF‐α and IL‐6 mRNA levels in cardiomyocytes isolated from sham and CHF rats. (**E**) TNF‐α and IL‐6 protein contents in cardiomyocytes isolated from sham and CHF rats (data are means ± SD, n = 4–6/group, *P < 0.05, **P < 0.01 *versus* respective sham).

Compared to sham‐operated rats, the mRNA and protein levels of TNF‐α and IL‐6 were increased in both the infarct and the remote myocardium of CHF rats, while comparable levels were observed between the infarct and remote areas (Fig. 2A and B). Circulating levels of TNF‐α and IL‐6 in CHF rats were increased as well (Fig. 2C). Notably, in cardiomyocytes isolated from CHF rats, the mRNA levels of both TNF‐α and IL‐6 were significantly higher than those in sham cardiomyocytes (Fig. 2D), while the protein contents of TNF‐α and IL‐6 were comparable between the sham and CHF cardiomyocytes (Fig. 2E). Although there was a tendency of increase in TNF‐α and IL‐6 proteins in CHF cardiomyocytes, no statistical significance was observed (Fig. 2E). This discrepancy might be attributable to the enzyme‐digestion process for isolating cardiomyocytes. Combined together, the above results clearly suggest the presence of myocardial and systemic inflammation in CHF.

### Increased TLR4 Expression in cardiomyocytes of the failing heart

**Figure 3:**
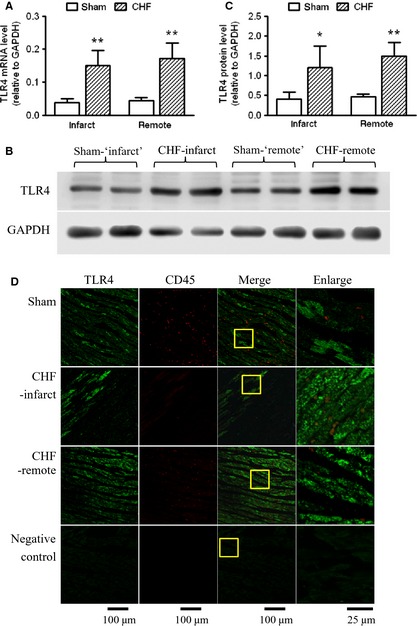
Increased toll‐like receptor 4 (TLR4) expression in the myocardium of chronic heart failure (CHF) rats. (**A**) TLR4 mRNA levels in infarct and remote myocardium of sham and CHF rats (n = 6/group). (**B**) Representative Western blot images and (**C**) quantification of TLR4 proteins in infarct and remote myocardium of sham and CHF rats (n = 4/group). (**D**) Representative immunohistochemistry images of heart sections stained with TLR4 (green) and CD45 (red). The yellow box indicates the enlarged area shown on the right (data are means ± SD, *P < 0.05, **P < 0.01 *versus* respective sham).

**Figure 4:**
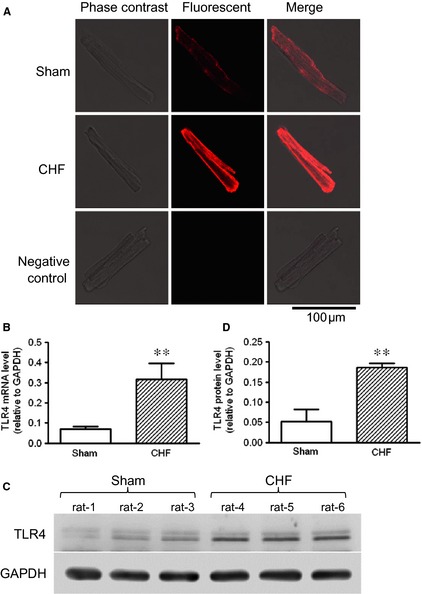
Increased toll‐like receptor 4 (TLR4) expression in the surviving cardiomyocytes of chronic heart failure (CHF) rats. (**A**) Representative immunofluorescent images of TLR4 in cardiomyocytes isolated from sham and CHF rats. (**B**) TLR4 mRNA levels in cardiomyocytes isolated from sham and CHF rats. (**C**) Representative Western blot images and (**D**) quantification of TLR4 proteins in cardiomyocytes isolated from sham and CHF rats (data are means ± SD, n = 6/group, **P < 0.01 *versus* sham).

As shown by real‐time RT‐PCR and Western blot analysis, TLR4 mRNA and protein levels were increased in both the infarct and the remote myocardium of CHF rats, while the infarct and remote areas are comparable (Fig. 3A–C). The immunohistofluorescence staining showed patches of TLR4‐positive signals in cardiomyocytes in heart sections from the sham‐operated rats. After 4 weeks of MI, more extensive and intense TLR4 signals were observed in cardiomyocytes in both the peri‐infarct and remote regions, suggesting increased expression of TLR4 (Fig. 3D). Consistent with this, the immunostaining of isolated cardiomyocytes also showed more intense signals of TLR4 in CHF myocytes, which were majorly localized on the cell surface, with relatively weak and regional distribution in cytosol (Fig. 4A). In contrast to the evident TLR4‐positive signals in cardiac muscle, leucocytes were mostly absent of TLR4 staining in both the sham and the CHF hearts, although they exhibited significant staining for a pan‐leucocyte marker CD45 (Fig. 3D). It is indicated that inflammatory cells infiltrating the myocardium may not express significant amounts of TLR4.

In accordance with the observation of increased TLR4 expression in heart tissue, increases of TLR4 mRNA and protein levels were observed in cardiomyocytes isolated from CHF rats (Fig. 4B–D). It is suggested that the surviving cardiomyocytes in post‐MI failing hearts express a greater amount of TLR4.

### Intra‐myocardial application of TLR4‐shRNA lentivirus reduced inflammation and improved heart function after MI

**Figure 5:**
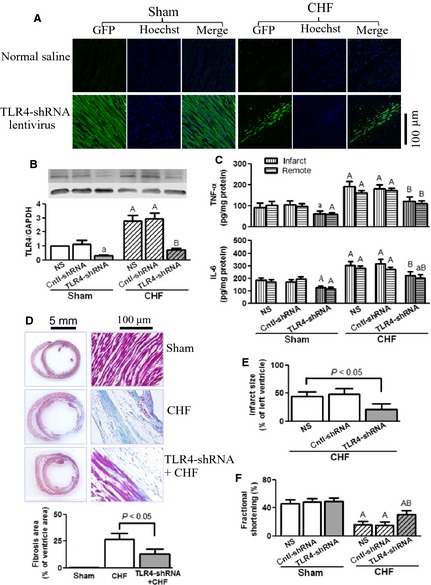
Toll‐like receptor 4 (TLR4)‐shRNA lentivirus reduced myocardial inflammation and improved heart function after myocardial infarction (MI). The rats received intra‐myocardial injection of normal saline (NS), control‐shRNA lentivirus or TLR4‐shRNA lentivirus (1 × 10^9^ TU/ml, 100 μl/heart) just after left anterior descending coronary artery (LAD) ligation or sham operation. All examinations were performed after 4 weeks of MI. (**A**) Expression of green fluorescent protein (GFP; green), the marker gene carried by TLR4‐shRNA lentivirus, in the myocardium. The nuclei were counter‐stained with Hoechst 33258 (blue). (**B**) Representative Western blot images and quantification of TLR4 proteins in sham and chronic heart failure (CHF) myocardium. (**C**) tumour necrosis factor (TNF)‐α and interleukin (IL)‐6 protein contents in infarct and remote myocardium. (**D**) Representative images of Masson's trichrome staining (upper panel) and quantification (lower panel) of post‐infarct failing hearts, showing that TLR4‐shRNA lentivirus reduced cardiac fibrosis. Cross‐sections were cut at the midhorizontal plane of the fixed paraffin‐embedded heart, and stained with Masson's trichrome reagents. (**E**) Infarct size of post‐infarct failing hearts. (**F**) Fractional shortening (%) of the left ventricle (data are means ± SD, n = 4/group, ^a^ *P* < 0.05, ^A^ *P* < 0.01 *versus* respective sham‐NS; ^B^ *P* < 0.01 *versus* respective CHF‐NS).

The intra‐myocardial injection of TLR4‐shRNA lentivirus resulted in a clear expression of green fluorescent protein (GFP), the marker gene carried by the lentivirus. In sham hearts receiving lentivirus, homogenous green fluorescence was observed in the myocardium. In CHF hearts, the expression of GFP was observed in both the infarct and border myocardium, suggesting the expression of TLR4‐shRNA (Fig. 5A). Western blot assay showed that TLR4‐shRNA reduced TLR4 protein levels by approximately 70%, in either sham or CHF rats (Fig. 5B). In The CHF rats receiving TLR4‐shRNA lentivirus, the increases of TNF‐α and IL‐6 production in the infarct and remote myocardium was significantly blunted (Fig. 5C). Concomitantly, myocardial fibrosis was reduced as shown by the Masson's trichrome stain (Fig. 5D)*,* the infarct sized was reduced (Fig. 5E) and the fractional shortening of the left ventricle was significantly improved (Fig. 5F). It is demonstrated here that the inhibition of TLR4 expression attenuated cardiac inflammation, reduced infarct size and improved heart function after infarction.

### Enhanced binding activity of TLR4 in CHF cardiomyocytes to LPS and HSP60

**Figure 6:**
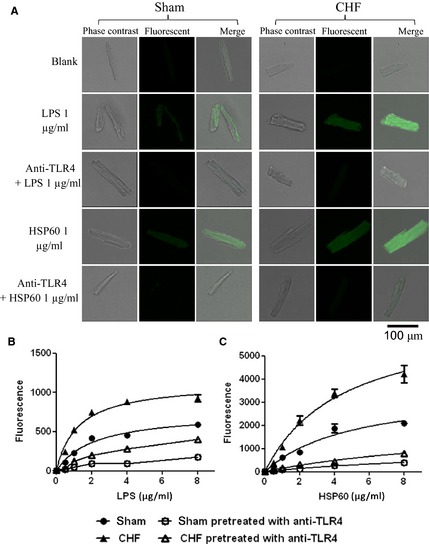
Enhanced binding activity of toll‐like receptor 4 (TLR4) in chronic heart failure (CHF) cardiomyocytes to lipopolysaccharide (LPS) and heat shock protein 60 (HSP60). Isolated cardiomyocytes were cultured in a CO_2_ incubator at 37°C for 24 hrs, then the binding assay was performed at 4°C for 30 min. To block TLR4, cultured cardiomyocytes were incubated with TLR4 neutralizing antibody (anti‐TLR4, 5 μg/ml) at 37°C for 15 min., and subsequently incubated with FITC‐LPS or OG‐HSP60 at 4°C for 30 min. (**A**) Representative fluorescent images of isolated cardiomyocytes after the incubation with FITC‐LPS (green) or OG‐HSP60 (green). (**B**) Binding curves of FITC‐LPS to cardiomyocytes. (**C**) Binding curves of OG‐HSP60 to cardiomyocytes.

Lipopolysaccharide and HSP60 are established ligands for TLR4 expressed in cardiomyocytes 15, 18. In accordance, the present study observed significant binding on the cultured cardiomyocytes when incubated with FITC‐LPS or OG‐HSP60 (Fig. 6A). The binding signals were more intense in CHF compared to sham cardiomyocytes. The pre‐incubation with TLR4 antibody reduced the binding of FITC‐LPS and OG‐HSP60 to either sham or CHF cardiomyocytes, suggesting that TLR4 mediated the binding (Fig. 6A). According to the binding curves (Fig. 6B and C), the bindings of FITC‐LPS and OG‐HSP60 to cardiomyocytes are saturable. In cardiomyocytes from sham rats, the *B*_max_ for FITC‐LPS and OG‐HSP60 was 741.6 ± 51.0 and 3559 ± 586.3, and the Kd was 2.1 ± 0.4 and 4.9 ± 1.6 μg/l respectively. In CHF cardiomyocytes, the *B*_max_ for FITC‐LPS and OG‐HSP60 significantly increased to 1121 ± 56.3 and 6748 ± 774.2 respectively (both *P* < 0.01 *versus* sham), whereas the *K*_D_ remained unchanged (1.2 ± 0.2 and 4.5 ± 1.0 μg/l, both *P* > 0.05 *versus* sham). It is suggested that the binding capacity of TLR4 to LPS and HSP60 was increased in CHF cardiomyocytes, though the binding affinity was not changed significantly. In addition, in both of the sham and CHF myocytes, the pre‐treatment with anti‐TLR4 antibodies (5–10 μg/ml) significantly inhibited the binding of FITC‐LPS and OG‐HSP60 (Fig. 6B and C), suggesting that FITC‐LPS and OG‐HSP60 were bound to TLR4.

### Increased inflammation mediated by TLR4 in CHF cardiomyocytes

**Figure 7:**
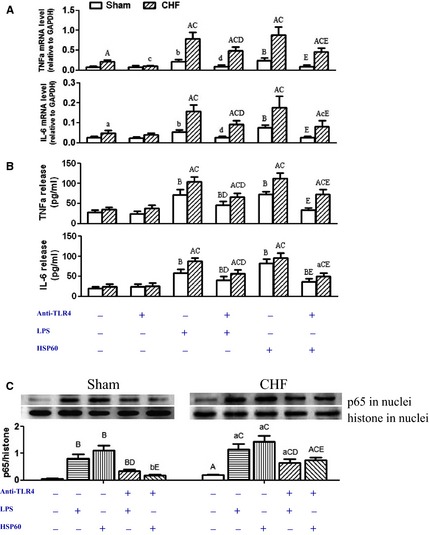
Increased cytokine production mediated by toll‐like receptor 4 (TLR4) in chronic heart failure (CHF) cardiomyocytes. Cultured cardiomocytes from sham and CHF rats were treated with lipopolysaccharide (LPS; 1 μg/ml) or heat shock protein 60 (HSP60; 1 μg/ml) for 6 hrs. TLR4 neutralizing antibody (anti‐TLR4, 5 μg/ml) was added 15 min before LPS or HSP60 treatment. (**A**) Tumour necrosis factor (TNF)‐α and interleukin (IL)‐6 mRNA levels (n = 6/group). (**B**) The amount of TNF‐α and IL‐6 released into culture supernatant (n = 6/group). (**C**) Representative Western blot images and quantification of p65 in the nuclei of cardiomyocytes from three independent experiments (data are means ± SD, ^a^ *P* < 0.05, ^A^ *P* < 0.01 *versus* respective sham; ^b^ *P* < 0.05, ^B^ *P* < 0.01 *versus* sham‐blank; ^c^ *P* < 0.05, ^C^ *P* < 0.01 *versus* CHF‐blank; ^d^ *P* < 0.05, ^D^ *P* < 0.01 *versus* respective LPS; ^e^ *P* < 0.05, ^E^ *P* < 0.01 *versus* respective HSP60).

We previously observed that LPS (1 μg/ml) and HSP60 (1 μg/ml) induced the mRNA expression and release of TNF‐α and IL‐6 in isolated adult rat cardiomyocytes, which reached a peak at 6 hrs 15. The present study used the same protocol of treatment, and observed similar increases in TNF‐α and IL‐6 production in sham cardiomyocytes (Fig. 7A and B). In CHF cardiomyocytes, the baseline level of TNF‐α and IL‐6 mRNA expression was higher than that in sham cardiomyocytes, while the baseline release amount of TNF‐α and IL‐6 was comparable. The treatment with either LPS or HSP60 resulted in two‐ to threefold increases in TNF‐α and IL‐6 mRNAs in sham cardiomyocytes. In contrast, the same treatment with LPS or HSP60 led to three‐ to fivefold increases in TNF‐α and IL‐6 mRNAs in CHF cardiomyocytes (Fig. 7A). Consistent with the mRNA responses, LPS and HSP60 induced more amount of TNF‐α and IL‐6 release into the culture medium in CHF cardiomyocytes (Fig. 7B). The pre‐incubation with anti‐TLR4 antibodies (5 μg/ml) significantly inhibited the pro‐inflammatory effects of LPS and HSP60 in both sham and CHF cardiomyocytes (Fig. 7A and B). However, the isotype control antibody (IgG, 5 μg/ml) had no effects on LPS or HSP60 (data not shown). Consistent with increased cytokine production, NF‐κB was activated by LPS and HSP60, as shown by the increases in nuclear accumulation of p65 (Fig. 7C). Also, the activation of NF‐κB was inhibited after blocking TLR4. Compared to sham cardiomyocytes, greater amounts of p65 proteins were observed in the nuclei of CHF cardiomyocytes, suggesting the activation of NF‐κB in CHF cardiomyocytes (Fig. 7C). These results showed that LPS and HSP60 induced more robust inflammation in CHF cardiomyocytes, which was mediated by TLR4.

## Discussion

The persistent increase of inflammatory cytokines in circulation represents a common feature of CHF, which is independent of the aetiology 1, 2. Although the failing heart has been acknowledged as a source for cytokine production, the role of cardiomyocytes remains unclear. Here, using a rat model of MI‐induced CHF, we investigated the expression and pro‐inflammatory function of TLR4 in the surviving cardiomyocytes. This study shows that: (*i*) In MI‐induced CHF, inflammatory cytokine levels are elevated in the serum and the heart, with comparable levels between infarct and remote areas; (*ii*) TLR4 mRNA and protein levels are both increased in CHF hearts, with comparable levels between infarct and remote areas; (*iii*) The injection of lentivirus shRNA against TLR4 into the infarcted heart decreased inflammatory cytokine production, reduced infarct size and improved heart function; (*iv*) TLR4 expression was increased in CHF cardiomyocytes, as demonstrated by enhanced immunostaining for TLR4 on cardiomyocytes in CHF heart sections, as well as increases of TLR4 mRNA and proteins in cardiomyocytes isolated from CHF hearts; (*v*) TLR4 on CHF cardiomyocytes displays higher binding capacity for both PAMP and DAMP ligands and (*vi*) both PAMP and DAMP ligands of TLR4 induce greater production of inflammatory cytokines in CHF cardiomyocytes, which is inhibited by TLR4 neutralizing antibodies.

As the most common cause of CHF, MI has been documented to induce extensive inflammation in the heart. Multiple studies, including this study, observed increases of inflammatory cytokine expression in both the infarct and remote myocardium after MI 20, 21, 22. It is readily understood that infarct inflammation may result from myocardial damage and healing stress, which involve immune cell infiltration 23, 24, 25. However, it is noteworthy here that the remote area remains in the inflammatory state after MI, which can be observed even after 7 weeks 26. The present study observed remote inflammation at 4 weeks. Monocyte/macrophage infiltration was shown to contribute to remote inflammation, but their effects may not last beyond 2 weeks after MI 27. In a recent review, we discussed several lines of data that indicate the ability of cardiomyocytes to be pro‐inflammatory cells 7. One of the data is that cardiomyocytes express a variety of PRRs including TLRs, whose activation induces innate immune responses, manifested as the activation of NF‐κB and inflammation 7. Toll‐like receptor 4 is a major subtype of TLRs expressed by cardiomyocytes 6. We previously observed that TLR4 mediates inflammation induced by short‐term ischaemia through recruiting MyD88, but not Trif, in a rat cardiomyocyte cell line, as well as rat myocardium 15. The present study showed the contribution of cardiomycyte TLR4 to inflammation after long‐term ischaemia. We observed increased expression and binding activity of TLR4 in cardiomycytes isolated from post‐infarct CHF hearts, which mediated stronger inflammatory responses to both PAMP and DAMP ligands. It is suggested that up‐regulated TLR4 expression and function cause cardiomyocytes to act as pro‐inflammatory cells in post‐infarct failing hearts.

This study, to our knowledge, is the first to address cardiomyocyte TLR4 after long‐term MI. We previously observed increases of TLR4 mRNA and protein in rat myocardium after 4 h of MI 15. Fallach *et al*. observed increased immunohistochemical staining for TLR4 in mice hearts at 4 hrs and 24 hrs after MI 14. In mice hearts after 4 days of MI, Frantz *et al*. observed enhanced and predominantly sarcolemmal staining in the border zone, and scattered foci of intense TLR4 staining in adjacent regions of contiguous cardiomyocytes in the remote zone. In contrast, the infiltrating inflammatory cells exhibited no labelling for TLR4 12. Differently, Timmers *et al*. observed positive TLR4 staining in both cardiomyocytes and inflammatory cells (macrophages), but no change in signal intensity compared to sham, in murine hearts on day 4 after MI; whereas no more TLR4 expressing macrophages were observed in the infarct area on day 28 28. The present study observed increased TLR4 staining in cardiomyocytes, but absence of TLR4 staining in infiltrating leucocytes, at 4 weeks after MI. There is a possibility that the leucocytes in the infarct area 4 weeks after MI induction are predominantly reparatory macrophages, which have no inflammatory phenotype and, therefore, do not express detectable TLR4 receptors 23. Despite the discrepancies, the above studies consistently recognized cardiomyocytes as a dominant cell type in the heart that expresses TLR4. In isolated cardiomyocytes, this study observed enhanced immunostaining for TLR4, as well as increased mRNA and protein levels of TLR4, after 4 weeks of MI. Functional study also showed enhanced inflammatory responses of cardiomyocyte TLR4 to PAMP and DAMP ligands. These results provide direct evidence for the up‐regulation of TLR4 expression and function after long‐term ischaemia.

Previous studies have demonstrated direct responses of TLRs on cardiomyocytes to PAMP and DAMP ligands 7. Among them, LPS from Gram‐negative bacteria is the canonical PAMP ligand for TLR4, and HSP60 is an ischaemia‐derived DAMP ligand for TLR4. Lipopolysaccharide has been widely used as a tool drug to activate TLR4. In isolated mouse cardiomyocytes, activation of TLR4 by LPS increases NF‐κB transcriptional activity, induces cytokine production and reduces myocyte contractility 29, 30. The present study observed increased binding capacity and pro‐inflammatory response of TLR4 to LPS in CHF cardiomyocytes, suggesting that TLR4 on CHF cardiomyocytes mediates pro‐inflammatory and cardiac depressive effects. The HSP60 is a DAMP molecule that can be actively secreted from ischaemic cardiomyocytes through specific pathways dependent on both lipid rafts and exosomes 31. Studies from other labs and ours have showed that ischaemia, either alone or followed by reperfusion, induces marked release of HSP60 from cardiomyocytes, which can activate TLR4 and induce cytokine expression in cardiomyocytes 15, 18, 31, 32. Furthermore, we found HSP60 in the circulation of post‐infarct CHF rats 16. The present study shows that TLR4 on CHF cardiomyocytes not only has higher binding capacity for HSP60, but also mediates more robust production of cytokines in response to HSP60. Taken together, it is conceivable that cardiomyocyte TLR4 might be activated by HSP60 in CHF circulation and trigger inflammation.

The role of cardiomyocyte TLR4 in CHF remains unclear. Most of the studies addressing cardiomyocyte TLR4 were performed in models of acute ischaemia/reperfusion. While multiple studies show that systemic deficiency of TLR4 alleviates myocardial inflammation and injury following acute ischaemia/reperfusion 7, 11, 33, controversies exist as to the causal role of cardiomyocyte TLR4 (*versus* leucocyte TLR4). In isolated perfused mouse heart subjected to global ischaemia and reperfusion, Ao *et al*. infused TLR4‐competent or TLR4‐defective neutrophils into TLR4‐competent or TLR4‐defective hearts during reperfusion, and observed that myocardial TLR4, rather than neutrophil TLR4, is the determinant of neutrophil infiltration after ischaemia 34. By using chimeric mice, Fallach *et al*. 14 and Avlas *et al*. 35 reported that cardiomyocyte TLR4, rather than leucocyte TLR4, plays a greater role in cardiac inflammation and dysfunction caused by either LPS or coronary artery ligation. In contrast, Tavener *et al*. reported that leucocyte TLR4, rather than cardiomyocyte TLR4, mediates cardiac damage response to LPS 36. The above studies made short‐term observations with discrepancies. Timmers *et al*. made a long‐term observation, in which systemic TLR4 defectiveness reduced LV remodelling and preserved systolic function without affecting infarct size, 28 days after MI 28. The present study focused on cardiomyocyte TLR4 after 28 days of MI. We observed that lentivirus‐mediated knockdown of cardiac TLR4 inhibited inflammation, reduced infarct size and improved heart function *in vivo*. In accordance, the blockade of TLR4 in isolated CHF cardiomyocytes prevented the overinduction of inflammation by the PAMP and DAMP ligands. It is indicated that cardiomyocyte TLR4 remains active over long‐term of MI. By studying *in vivo* myocardium and isolated cardiomyocytes, we provide direct evidence that the increased expression and function of TLR4 on cardiomyocytes mediate inflammation and aggravate heart failure after MI.

Cardiomyocytes are not readily perceived as having an active role in inflammatory responses as they are muscle cells rather than immune cells. In the surviving cardiomyocytes of failing hearts, we observed increased mRNA and protein levels of TLR4, as well as enhanced binding capacities and pro‐inflammatory responses to PAMP and DAMP ligands. These observations, together with the previous finding that the DAMP ligands such as HSP60 can be released from ischaemia‐stressed cardiomyocytes through active mechanisms 7, strongly suggest that cardiomyocytes play an active and initial role in mediating inflammation after MI. The role of cardiomyocytes as an active source of inflammatory cytokines may not be neglected under CHF conditions.

## Conflicts of interest

None.
